# Knowledge of health effects and determinants of psychoactive substance use among secondary school students in Sokoto Metropolis, Nigeria

**DOI:** 10.11604/pamj.2021.40.109.22000

**Published:** 2021-10-19

**Authors:** Auwal Usman Abubakar, Aisha Ahmad Abubakar, Mu'awiyya Babale Sufiyan, Muhammad Shakir Balogun, Kehinde Joseph Awosan, Ismail Abdullateef Raji, Aishat Bukola Usman, Abdulkarim Mohammad Abdullahi, Ahmad Muhammad Njidda, Hashim Abdulmumin Bala, Aminu Umar Kaoje, Patrik Mboya Nguku, Adamu Usman Shehu

**Affiliations:** 1 Nigerian Field Epidemiology and Laboratory Training Program, Nigeria,; 2 Department of Community Medicine, Usmanu Danfodiyo University Teaching Hospital, Sokoto, Nigeria,; 3 Department of Community Medicine, Ahmadu Bello University, Zaria, Nigeria,; 4 Department of Community Medicine, Gombe State University, Gombe, Nigeria

**Keywords:** Knowledge, prevalence, determinants, psychoactive substance use, Sokoto, Nigeria

## Abstract

**Introduction:**

psychoactive substance use (PSU) is a patterned use of a drug in which the user consumes the substance in amounts or methods which are harmful to themselves or others. Psychoactive substance use takes a considerable toll on financial status, academic achievement and health status of addicts. In Nigeria, PSU is on the increase, one of the most disturbing health-related problems and a leading cause of premature death among school aged population worldwide. We therefore, determined the knowledge of health effects and determinants of psychoactive substance use among secondary school students in Sokoto Metropolis, Nigeria.

**Methods:**

we conducted a cross-sectional study among 430 secondary school students that were selected using multistage sampling in Sokoto, Northwestern, Nigeria from April to May 2019. We collected data using a semi-structured, interviewer-administered questionnaire. We calculated proportions and adjusted odds ratios (OR) with 95% confidence intervals (CI) in a binary logistic regression model.

**Results:**

knowledge of health effects of PSU was good in 38.1% of the respondents with a mean score of 19.6 ± 10.0. The overall prevalence of PSU was high among current users (16.3%), male participants (78.6%) and those aged 17-years or more (68.6%). Independent predictors of current use of psychoactive substances were poor knowledge of health effects (aOR: 4.1, 95% CI: 1.7-10.0) and father´s use of psychoactive substances (aOR: 10.3, 95% CI= 1.9-57.1).

**Conclusion:**

knowledge of health effects of psychoactive substances was generally poor among the participants with an associated high prevalence among current users. Poor knowledge of its health effects determines the use of psychoactive substances. We conducted awareness campaigns and health talk on health effects of PSU to secondary school students in the State. The Federal Ministry of Education should ensure that PSU-related topics are incorporated in the secondary school curriculum.

## Introduction

Psychoactive substance use constitutes a major public health and social problem worldwide, with alcohol, tobacco and marijuana being the most commonly used [1]. These drugs take a huge toll not just financially, but in lost potential in academic achievement, poor health and untimely death [2]. Globally, over 29 million people suffer from drug use disorders, and of those, 12 million are people who inject drugs (PWID) of whom 14.0% are living with HIV [3]. In developing countries, recent trends indicate that the use of psychoactive substances (PASs) has dramatically increased [4]. Studies have shown a rise in consumption, as well as early initiation, increasing female involvement, and a trend towards multiple substance use among adolescents in Nigeria [5]. Epidemiological data from school surveys in Nigeria have shown that substance abuse is common with a rising prevalence and a decreasing age of onset. It is also one of the most disturbing health-related problems among youths [5]. Most often the adolescents and young individuals start by experimenting with the so-called “gateways drugs” such as tobacco, alcohol and later progress to more dangerous ones such as marijuana and cocaine [5,6].

Police reports revealed that cannabis, and recently codeine and shisha are available on the Nigerian streets of urban areas [7]. Although to a lesser extent cocaine and psychostimulants are also known to be abused in this country [7]. In Birnin Kebbi North-Western Nigeria, lifetime prevalence and current prevalence of tobacco smoking were 32.2% and 20.8% respectively [8]. Literature suggests that peer influence, family factors and personal background play an important role in the development of youth substance use behavior [9]. It is not rare for Nigerian secondary school students to consume alcoholic drinks; this consumption could be due to their curiosity as adolescents, an irresistible urge, emotional disturbances such as anxiety, the subculture, and the influence of advertisements [10]. Young people such as secondary school students particularly those who consume psychoactive substance are more prone to engaging in high-risk behavior, which include unprotected sex, early sexual debut or sex with multiple partners, in addition to strong relation to perceived academic achievements [11]. Abuse of drugs not only holds back the economy but is also a blow to the country as its youths become less productive [7]. Adolescents´ knowledge of health effects of psychoactive substance use is very fundamental when undertaking research aimed at psychoactive substance cessation interventions. However, the extent of consumption among secondary school students and their understanding of its effects on human health is poorly documented in many Nigerian States including Sokoto [10]. This study was therefore conducted to assess the knowledge of health effects and determinants of psychoactive substance use among secondary school students in Sokoto Metropolis, Nigeria.

## Methods

**Study area:** this study was conducted in Sokoto Metropolis the capital of Sokoto State in North-Western Nigeria. The metropolis comprises Sokoto North, Sokoto South and some parts of Dange-Shuni and Wamakko local government areas (LGA). There are 111 secondary schools (76 public and 35 private schools) in the State´s metropolitan LGAs, including the two federal government-owned colleges. The total adolescent population in the State is 1,118,787 as projected from 2006 census. The majority of the schools operate between 7:30 am to 1:30 pm. The three tertiary health institutions in the State; the Usmanu Danfodiyo University Teaching Hospital Sokoto, specialist hospital Sokoto and Federal Neuro-psychiatric Hospital in Kware offers rehabilitation services for psychoactive substance/drug related addiction in the State. In addition, the Sokoto State office of the national drug law enforcement agency also offers counseling on substance use and misuse and rehabilitation services for the affected individuals.

**Study design and population:** a cross-sectional study was conducted among secondary school students from April to May 2019. Only the students in public and private secondary schools registered by the government (as indicated in the list obtained from the state ministry of basic education board) were considered eligible. Those who were not regular students (students on excursion, external Senior Secondary Certificate of Education (SSCE) candidates) were excluded.

**Sample size estimation and sampling technique:** the sample size was estimated using the statistical formula for calculating the sample size for descriptive studies [3].


$$n=Z\alpha^{2}pq/d^{2}$$

With a 50.7% expected prevalence (p) of psych oactive substance use from a previous study, [6] a precision level (d) of 5%, and an anticipated 90% response rate. A total of 430 students were ultimately enrolled into the study. The eligible participants were selected using a multi-stage sampling technique. At the first stage, 2 of 4 metropolitan LGAs - Sokoto South and Wamakko - were randomly selected by balloting. At the second stage, the schools were stratified into public and private, line listing of all the public and private schools in the selected LGAs was done, and 12 (8 public and 4 private) of 27 (19 public and 8 private) schools were randomly selected by balloting using equal allocation of 6 (4 public and 2 private) schools from Wamakko and Sokoto South LGAs respectively. At the third stage, students were stratified into junior and senior classes, line list of all students was obtained from the respective schools and proportionately allocated. However, systematic sampling technique (with replacement) was used to recruit study participants from the respective schools.

**Data collection and analysis:** a pretested structured interviewer-administered questionnaire was used to collect data on the respondents´ socio-demographic characteristics, knowledge of health effects of psychoactive substance use, prevalence and pattern of psychoactive substance use. The questions were adapted from different validated questionnaires [2,4,12]. The face and content validity were carried out by the principal researcher´s supervisors who are expert in the field, whereas the test-retest reliability was conducted to establish the reliability of the instrument where a reliability coefficient of 0.812 was achieved. The questionnaire was pretested on 43 secondary school students in Sokoto North LGA, and appropriate corrections were made based on the deficiencies detected in the instrument during the pretesting. Four unemployed graduates and two undergraduates of tertiary institutions in Sokoto State were trained and assisted the researcher in data collection. The training focused on the conduct of research including research ethics, objectives of the study, selection of study participants and use of the survey instrument. Knowledge section consists of 39 questions. Correct response on knowledge of health effects of psychoactive substances was scored one mark, while incorrect response was scored zero and the aggregate scores for each respondent were reported in proportions. Knowledge scores ≥ 23.4 (≥ 60%) and < 23.4 (< 60%) were graded as good and poor knowledge respectively [13]. Descriptive statistics analysis such as mean and standard deviation were done for continuous variables whereas, categorical data were presented in frequencies and proportions. Chi-square and Fisher´s exact test were also performed to determine the association between independent and dependent variables whereas binary logistic regression model was used to identify factors that predicted psychoactive substance use among users. IBM Statistical Package for the Social Sciences (SPSS) version 23 software was used for the analysis. All levels of significance were set at p < 0.05.

**Ethical consideration:** institutional ethical clearance was obtained from the Ethical Committee of Sokoto State Ministry of Health, Sokoto, Nigeria (SMH/1580/V.IV). Permission to conduct the study was obtained from the Honorable Commissioner, Ministry of Education, Sokoto State, Nigeria. In addition, a written assent was obtained from students below the age of 18 years and consent was also sought from the principal/guardian or parents as the case may be. Participants were assured of strict confidentiality of their responses and informed that their participation is voluntary and would incur no loss if they refuse to participate in the study.

## Results

A total of 430 participants were recruited to participate in the study and all completed the interview, giving a response rate of 100%. Their mean age was 16.3 ± 3.1 years. Most of the respondents were males [284 (68.4%)], Hausa [253 (58.6%)] by ethnicity and attending single-sex schools 229 (53.3%). A majority [44 (80.0%)] of the respondents were day students, from monogamous family setting [235 (54.7%)] and of socio-economic class II [122 (28.4%)] (Table 1). One hundred and sixty-four respondents (38.1%) had good knowledge of the health effects of psychoactive substance use with the mean knowledge score of (19.6 ± 10.0). Almost half of the respondents knew poor concentration [211 (49.1%)] and inability to sleep (49.3%) as psychological health effect of psychoactive substance use. Unprotected premarital sex [136 (31.6%)] was the least known social health effect of psychoactive substance use among the respondents (Table 2).

**Table 1:** socio-demographic characteristics and family background of respondents

Variables		Frequency n = 430	Percent
**Age group (Years)**			
	10-12	51	11.9
	13-15	129	30.0
	16-18	148	34.4
	≥ 19	102	23.7
**Sex**			
	Male	294	68.4
	Female	136	31.6
**Ethnicity**			
	Hausa	253	58.8
	Fulani	42	9.8
	Yoruba	72	16.7
	Igbo	32	7.4
	Others	31	7.2
**Religion**			
	Muslim	352	81.9
	Christian	78	18.1
**School attendance**			
	Public	301	70.0
	Private	129	30.0
**Composition of school attended**			
	Single sex school	229	53.3
	Co-education school	201	46.7
**Class**			
	Junior class	158	36.7
	Senior class	272	63.3
**Type of student**			
	Day student	344	80.0
	Boarding student	86	20.0
**Family background**			
	Monogamous	235	54.7
	Polygamous	195	45.3

Others = Zabarmawa, Dakkarawa

**Table 2:** respondents’ knowledge of health effects of psychoactive substance use

Variables		Frequency n = 430	Percentage
**Knowledge of physical health effect of psychoactive substance use**			
	Knew hypertensive heart disease as physical health effect of PAS use	229	53.3
	Knew that liver and brain damage as physical health effects of PAS use	257	59.8
	Knew stroke as physical health effect of PAS use	204	47.4
	Knew headache as physical health effect of PAS use	247	57.4
	Knew irritability as physical health effect of PAS use	161	37.4
	Knew interference with speech as physical health effect of PAS use	187	43.5
	Knew interference with balance/ gait as physical health effect of PAS use	148	34.4
	Knew weight loss as physical health effect of PAS use	156	36.3
	Knew premature death as physical health effect of PAS use	138	32.1
	Knew diseases of the lungs as physical health effect of PAS use	167	38.8
	Knew inability to carry pregnancy to term as physical health effect of PAS use	77	17.9
	Knew HIV/AIDS as physical health effect of PAS use	68	15.8
	Knew accident as physical health effect of PAS use	229	53.3
	Knew hand tremor as physical health effect of PAS use	113	26.3
**Knowledge of psychological health effect of psychoactive substance use**			
	Knew depression as psychological health effect of PAS use	300	69.8
	Knew anxiety as psychological health effect of PAS use	261	60.7
	Knew restlessness as psychological health effect of PAS use	215	50.0
	Knew poor concentration as psychological health effect of PAS use	211	49.1
	Knew inability to sleep as psychological health effect of PAS use	212	49.3
	Knew emotional problem as psychological health effect of PAS use	176	40.9
	Knew mental illness as psychological health effect of PAS use	292	67.9
**Knowledge of social health effect of psychoactive substance use**			
	Knew psychoactive substance use alter one's financial status	217	50.5
	Knew unprotected premarital sex as social health effect of PAS use	136	31.6
	Knew raping as social health effect of PAS use	250	58.1
	Knew kidnapping as social health effect of PAS use	251	58.4
	Knew psychoactive substance use alter family or friends peer relationship	184	42.8
	Knew PAS use make one commit suicide	234	54.4
	Knew psychoactive substance use make one violent	230	53.5
	Knew psychoactive substance use lead one into robbery	226	52.6
	Knew psychoactive substance use lead one into cultism	197	45.8
	Knew psychoactive substance use lead one into terrorism	212	49.3
**Knowledge of intellectual health effect of psychoactive substance use**			
	Knew poor memory as intellectual health effect of PAS use	309	71.9
	Knew poor coordination as intellectual health effect of PAS use	213	49.5
	Knew poor academic performance as intellectual health effect of PAS use	283	65.8
	Knew poor reasoning as intellectual health effect of PAS use	267	62.1
	Knew poor judgement as intellectual health effect of PAS use	255	59.3
**Knowledge of intellectual health effect of psychoactive substance use**			
	Knew security threat as environmental health effect of PAS use	310	72.1
	Knew damage to properties as environmental health effect of PAS use	301	70.0
	Knew environmental pollution environmental health effect of PAS use	302	70.2
**Overall knowledge grading**			
	Poor knowledge	266	61.9
	Good knowledge	164	38.1

PAS = Psychoactive substance, Good knowledge = knowledge score ≥ 60%

**Figure 1:**
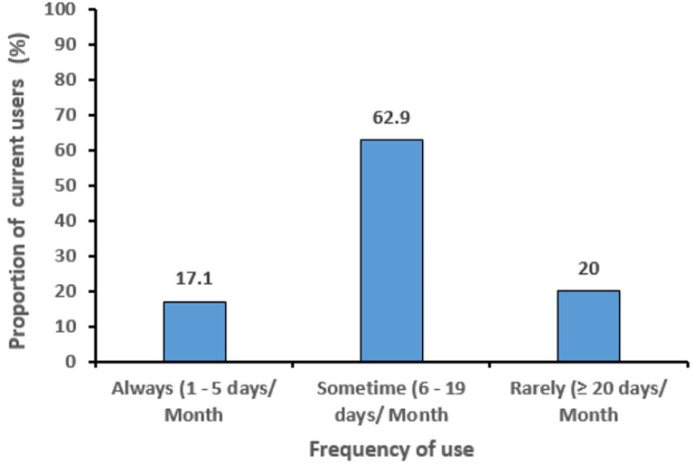
frequency of psychoactive substance use among current users (n=70)

The overall prevalence of psycho-active substance use among the respondents was 16.3%. A majority [33 (47.1%)] of the respondents claimed they used psychoactive substances to reduce stress, and to be acceptable to their friends [32 (45.7%)]. Whereas 14 (28.6%) and 22 (31.4%) respondents claimed to have used psychoactive substances as a result of peer pressure and to be more influential among their peer group respectively. Most of the current psychoactive substance users were male [55 (78.6%)] and aged 17 years or more [48 (68.6%)]. In relation to substance types, tobacco [23 (41.8%)] was the commonest and more frequent among males. While shisha [8 (53.3%)] was the most frequently consumed substance among females (Table 3). Most [44 (62.9%)] of those that currently used psychoactive substance, used it at least once every 6 to 19 days (Figure 1). Secondary school students who had poor knowledge of psychoactive substance were more likely to be current users compared to those with good knowledge (aOR: 4.1; 95% CI= 1.7-10.0). Also, those whose fathers used one or more psychoactive substance were more likely to be current users compared to those whose fathers did not use any substance (aOR: 10.3; 95% CI= 1.9-57.1) (Table 4).

**Table 3:** pattern of psychoactive substance use by sex among current users

Variables		Sex		
		Male	Female	Total
		n = 55	n = 15	n = 70
		Freq (%)	Freq (%)	Freq (%)
Type of substance				
	Tobacco	23 (41.8)	1 (6.7)	24 (34.3)
	Alcohol	2 (3.6)	4 (26.7)	6 (8.6)
	Shisha	21 (38.2)	8 (53.3)	29 (41.4)
	Caffeine containing energy drink	20 (36.3)	6 (40.0)	26 (37.1)
	NSAID with caffeine	6 (10.9)	2 (25.0)	8 (11.4)
	Kolanut	9 (16.4)	2 (13.3)	11 (15.7)
	Cannabis	12 (21.8)	3 (13.3)	15 (21.4)
	Codeine	17 (30.9)	5 (33.3)	22 (31.4)
	Tramadol	10 (18.2)	0 (0.0)	10 (14.3)
	Others	7 (12.7)	1 (6.7)	8 (11.4)
	

Others = Rohypnol, Cocaine, Diazepam, Chaku Basir

**Table 4:** predictors of psychoactive substance use among current users

Variables	cOR (95% CI)	aOR (95% CI)
**Age** (< 15 years / ≥ 15 years*)	0.6 (0.2 - 1.4)	1.1 (0.3 - 5.0)
**Gender** (Male /Female*)	2.1 (1.0- 4.9)	0.5 (0.2 - 1.2)
**Type of school by ownership** (public / private*)	0.5 (0.2 - 1.3)	2.9 (0.6 - 14.4)
**Type of school by gender** (Single sex school / Co-educational*)	0.9 (0.5 - 2.0)	0.9 (0.3 - 2.9)
**Class (**Junior class / Senior class*)	0.8 (0.4 - 1.8)	0.6 (0.1 - 2.1)
**Knowledge grade** (Poor / Good*)	3.3 (1.6 - 10.0)	4.1 (1.7 - 10.0)
**Family background** (Monogamous / Polygamous*)	2.5 (1.1 - 5.0)	1.9 (0.9 - 4.0)
**Father uses one or more PAS** (Yes / No*)	5.0 (1.1 - 10.0)	10.3 (1.9 - 57.1)

## Discussion

This study assessed knowledge of health effects and determinants of psychoactive substance use among secondary school students in Sokoto metropolis, Nigeria. More respondents in this study were males. The sex distribution in this study is consistent with those obtained in several studies in Nigeria and abroad [7,8,14,15]. The preponderance of males in Nigerian secondary schools could be explained by the societal culture and attitude, especially in the northern part of the country that seems to favour the male child [3]. However, female child education has been on the rise not only in southern but also in the northern part of Nigeria [16]. This study demonstrates the knowledge of health effects of psychoactive substance to be good in at least a third of the respondents (38.1%) with a mean knowledge score of 19.6 ± 10.0. This finding is much lower compared to what was reported in Owerri, Southeastern Nigeria, Tanzania and India where they reported good knowledge of health effects of psychoactive substance use among students as 63.1%, 98.5% and 84.6% respectively [7,17,18]. Finding in this study could be as a result of scores obtained by the respondents in different sub-section questions that addressed knowledge of health effects of psychoactive substance use. For example, on knowledge of physical health effect, slightly above half of the respondents knew accident and hypertensive heart disease as physical health effects of psychoactive substance use as against 70.7% and 58.5% reported by Gotsang *et al*. in Botswana for traffic accident and heart diseases respectively [19]. Regarding knowledge of psychological health effects only about two-thirds of the respondents knew mental illness as psychological health effect as against 74.9% reported in Botswana [19].

The current PAS use prevalence of 16.3% was high in this study; this highlights the importance that any drug abuse preventive measures, formal policies on drug abuse need to be implemented much earlier in the student´s academic lives to achieve desired objectives. The finding is slightly lower than 20.8% reported by Gana *et al*. in Birnin Kebbi North-Western Nigeria and 25.0% reported by Dasgupta *et al*. in India [8,18]. But much higher than the 9.4% reported by Shehu *et al*. in Zaria for the prevalence of marijuana, [20] and 8.5% reported by Mosibo *et al*. in Dodoma Municipality, Tanzania [7]. The finding was much lower than the 47.9% reported by Birhanu *et al*. in Woreta Town, North-western Ethiopia for the current prevalence of substance use [21]. These differences could be as a result accessibility of the type of substance studied, or it could be as a result of disparities in methodologies and socio-cultural characteristics of the respondents. In this study, the distribution of current users of psychoactive substance was disproportionate according to gender, where more than two-thirds were males. However, these findings were comparable to the findings of studies by other authors, where similar results were noted [1,14]. This may be because males are more likely to be adventurous than their female counterparts and they are more likely to be experimenting during their adolescent years. Females, on the other hand, enjoy more supervision of parents/guardian because of the fear that their engagement in social activities could predispose them to being wayward [22].

The most frequently used substance among male respondents were tobacco and shisha, while the least used being cocaine and diazepam. Whereas, among female current users, the most frequently used substances were shisha and energy drink, while the least consumed being Kolanut and Rohypnol; this contrasts with the finding in a study by Mosibo *et al*. who reported alcohol use 47.1% as the most frequent and codeine 5.9% and heroine 5.9% as the least frequent among males [7]. This indiscriminate use of most frequently used substance may partly result from easy access and availability. Over the counter (OTC) purchases being a common phenomenon in Nigeria as compared to least used psychoactive substance that are mostly controlled drugs. Some of the reasons given by our respondents for psychoactive substance use were to reduce stress, be acceptable to friends, increase pleasure of life, enhance work performance, admiration by others and become more influential among peers. These were similar to findings reported in Owerri South-Eastern Nigeria, Botswana and India for initiation of substance use which included peer influence, to relieve stress, to increase pleasure of life and to be like their friends/relatives/role model [17-19].

A majority of the users in this study used a substance at least once every 6 to 19 days in a month. Whereas, less than a quarter (17.1%) were using them between 1-5 days in a month. Similar though much lower frequency of use was reported by Gana *et al*. in Birnin Kebbi where almost a quarter (23.6%) of smokers smoked cigarettes for more than five days during the previous month; [8] this highlights the rate at which adolescents are beginning to imbibe psychoactive substance use as a way of life. Factors found to predict current use of psychoactive substances in this study were respondent´s knowledge of health effects and father´s use of psychoactive substances. Shehu *et al*. in Zaria reported family background as a factor that influenced marijuana smoking. Contrary to the finding in this study, he reported peer pressure and attendance of social functions as factors that also influence marijuana smoking [20]. The strengths of the study lie in its ability to determine that the knowledge of health effect of psychoactive substance use was barely good in only about a third of the respondents. It also provides evidence-based information on the point prevalence as well as the pattern of psychoactive substance use among secondary school students that could be used in recommending to the ministry of education how to address this menace. A major weakness in this study is the inability to explore some of the emerging psychoactive substances mentioned by the respondents as others, for example, Chaku Basir. These substances could have been known in greater details if we had included qualitative techniques in the study design. Also, due to the sensitive nature of issues associated with the study, some of the respondents refused to open up, we minimised this by educating the study participants on the importance of the study in addition to informing them that the information given cannot be traced back to them and reassurances about confidentiality of information given by them.

## Conclusion

Knowledge of health effects of psychoactive substances was generally poor among secondary school students in Sokoto State. The prevalence of psychoactive substance use was high among them. The poor knowledge of health effects and having a father using psychoactive substances were predictors of psychoactive substance use among the study participants. Sokoto State ministry of basic and secondary education in collaboration with State ministry of health should provide periodic awareness campaigns on health effects of psychoactive substance use to all secondary school students in the State. The federal ministry of education should consider inclusion of psychoactive substance related topics in secondary school curriculum.

### What is known about this topic

- Psychoactive substance use is one of the most disturbing health-related problems and a leading cause of premature death among school aged population worldwide;
- Epidemiological data from school surveys in Nigeria have shown that substance abuse is common with a rising prevalence and a decreasing age of onset;
- Psychoactive substance use takes a considerable toll on financial status, academic achievement and health status of addicts.

### What this study adds

- This study has given an insight into the level of knowledge of health effects of psychoactive substances among secondary school students in Sokoto Metropolis, Northwestern Nigeria;
- The study has shown that there is high level of psychoactive substance use among secondary school students in Sokoto Metropolis, Northwestern Nigeria;
- This study found that poor knowledge of health effects and having a parent that uses psychoactive substances were predictors of psychoactive substance use among the study participants.
